# The *FKBP5* Gene Affects Alcohol Drinking in Knockout Mice and Is Implicated in Alcohol Drinking in Humans

**DOI:** 10.3390/ijms17081271

**Published:** 2016-08-05

**Authors:** Bin Qiu, Susan E. Luczak, Tamara L. Wall, Aaron M. Kirchhoff, Yuxue Xu, Mimy Y. Eng, Robert B. Stewart, Weinian Shou, Stephen L. Boehm, Julia A. Chester, Weidong Yong, Tiebing Liang

**Affiliations:** 1 Institute of Laboratory Animal Science, Chinese Academy of Medical Sciences (CAMS), Peking Union Medical College (PUMC), Beijing 100021, China; qiub@cnilas.org (B.Q.); xuyuxue1127@gmail.com (Y.X.); 2 Department of Psychology, University of Southern California, Los Angeles, CA 90089, USA; luczak@usc.edu; 3 Department of Psychiatry, University of California, San Diego, CA 92037, USA; twall@ucsd.edu; 4 Psychology Service, Veterans Affairs San Diego Healthcare System, San Diego, CA 92161, USA; 5 Veterans Medical Research Foundation, San Diego, CA 92161, USA; mimyeng@stanfordalumni.org; 6 Immunology and Microbial Science Department, Research Technician, The Scripps Research Institute, Scripps Clinic South Driveway, La Jolla, CA 92037, USA; amkirchhoff@gmail.com; 7 Department of Psychology, Indiana University-Purdue University Indianapolis, Indianapolis, IN 46202, USA; rstewart@iupui.edu (R.B.S.); slboehm@iupui.edu (S.L.B.); 8 Departments of Pediatrics and Medicine, Indiana University School of Medicine, Indianapolis, IN 46202, USA; wshou@iu.edu; 9 Department of Psychological Sciences, Purdue University, West Lafayette, IN 47907, USA; jchester@psych.purdue.edu; 10 Department of Medicine, Indiana University School of Medicine Gatch Hall, Indianapolis, IN 46202, USA

**Keywords:** *Fkbp5* knockout, alcohol drinking behavior, human alcohol use disorder

## Abstract

*FKBP5* encodes FK506-binding protein 5, a glucocorticoid receptor (GR)-binding protein implicated in various psychiatric disorders and alcohol withdrawal severity. The purpose of this study is to characterize alcohol preference and related phenotypes in *Fkbp5* knockout (KO) mice and to examine the role of *FKBP5* in human alcohol consumption. The following experiments were performed to characterize *Fkpb5* KO mice. (1) *Fkbp5* KO and wild-type (WT) EtOH consumption was tested using a two-bottle choice paradigm; (2) The EtOH elimination rate was measured after intraperitoneal (IP) injection of 2.0 g/kg EtOH; (3) Blood alcohol concentration (BAC) was measured after 3 h limited access of alcohol; (4) Brain region expression of *Fkbp5* was identified using LacZ staining; (5) Baseline corticosterone (CORT) was assessed. Additionally, two SNPs, *rs1360780* (C/T) and *rs3800373* (T/G), were selected to study the association of *FKBP5* with alcohol consumption in humans. Participants were college students (*n* = 1162) from 21–26 years of age with Chinese, Korean or Caucasian ethnicity. The results, compared to WT mice, for KO mice exhibited an increase in alcohol consumption that was not due to differences in taste sensitivity or alcohol metabolism. Higher BAC was found in KO mice after 3 h of EtOH access. *Fkbp5* was highly expressed in brain regions involved in the regulation of the stress response, such as the hippocampus, amygdala, dorsal raphe and locus coeruleus. Both genotypes exhibited similar basal levels of plasma corticosterone (CORT). Finally, single nucleotide polymorphisms (SNPs) in *FKBP5* were found to be associated with alcohol drinking in humans. These results suggest that the association between *FKBP5* and alcohol consumption is conserved in both mice and humans.

## 1. Introduction

Alcohol use disorder displays a high level of comorbidity with several psychiatric disorders [1,2]. Dysregulation of a primary component of the stress response, the hypothalamic-pituitary-adrenal (*HPA*) axis, has been implicated in the pathophysiology of these psychiatric disorders [3,4,5,6] and mediates the transition from episodic drug and alcohol use to dependence [7,8,9,10]. Glucocorticoids, glucocorticoid receptor (GR) and its binding proteins (e.g., Fkbp5, FK506 binding protein 5, also known as FKBP51) are critical *HPA* axis regulatory elements.

Diverse lines of research suggest a correlation between glucocorticoid levels and alcohol consumption. For instance, increases in *HPA* axis responsivity in young primates predicted higher levels of future alcohol consumption [11]. Alcohol consumption and withdrawal have both been shown to increase circulating glucocorticoids and to decrease GR availability [12], and the release of high levels of glucocorticoid peptides has been shown to sensitize the reward pathways in the brain [13,14,15]. Other research indicates that the GR plays an important role in the determination of alcohol abuse. Polymorphisms in the GR are associated with the onset of alcohol abuse in adolescents [6]; GR-mediated plasticity increased voluntary alcohol consumption in rats [16]; and a GR antagonist reduced alcohol intake in rats [17]. Thus, the GR is a worthy target of research aimed at identifying novel treatment strategies for alcohol use disorder.

GR binding proteins are critical for GR function and GR-regulated behavior. However, limited research has been done to investigate the role of GR binding proteins in behaviors related to alcohol use disorder. FKBP5 is a GR binding protein that acts as a co-chaperone of heat shock protein 90 and is involved in regulating GR activity, nuclear translocation and transcriptional regulation of GR-targeted genes [18,19,20]. Functionally, FKBP5 is a potent inhibitor of GR activation and a determinant of *HPA* axis regulation [21]. As replicated in other studies, both *Fkbp5* knockout (KO) mice and *FKBP5* knockdown neuronal cell cultures exhibit elevated GR nuclear translocation [22,23].

High expression of *Fkbp5* has been observed in the brain, especially, hippocampus [24], which is consistent with its function in the stress response. Increased hippocampal expression of *Fkbp5* mRNA, as well as increased plasma corticosterone (CORT) levels and adrenal gland weight were observed after chronic social defeat in mice [25]. When compared to WT mice prior to chronic social defeat, *Fkbp5*-deficient mice exhibited lower basal CORT and lower adrenal weights [26].

*Fkbp5* gene expression is not only induced by stress [27], but also by alcohol [28,29] and drug administration [30]. In addition, glucocorticoid treatment increased *Fkbp5* expression in peripheral tissue [31] and the brain [32]. Consistent with the effects of alcohol on *Fkbp5* gene expression in the CNS following acute alcohol injection, these findings suggest that increases in *Fkbp5* expression following steroid receptor activation reduce GR sensitivity and, in turn, modulate GR-related behavior [28,33].

*FKBP5* SNPs and gene expression levels are associated with the onset of posttraumatic stress disorder (PTSD) and anxiety disorder in humans [20,34]. Particularly, two SNPs, *rs1360780* (C/T) and *rs3800373* (T/G) are often used for association studies. In humans, alcohol use disorder is often comorbid with anxiety and other psychiatric disorders [2,35]. In rodents, anxiety is correlated with alcohol preference in various models of alcohol use disorder [36,37]. Genetic variations of *FKBP5* are associated with an increased risk for depression [38,39], PTSD [40] and bipolar disorder [41] and are also associated with a greater risk for comorbid alcohol dependence and PTSD onset [42]. Moreover, *FKBP5* SNPs are associated with the degree of cortisol response [20,21], response to antidepressants [38,39,40], heroin addiction [43] and alcohol withdrawal severity [5]. For example, heterozygous and homozygous carriers of the *rs3800373* (T/G and G/G) or *rs1360780* (C/T and T/T) variants are more likely to respond to antidepressant drugs [44]. Some findings suggest that the G allele of *rs3800373* and the T allele of *rs1360780* (minor alleles) may represent protective alleles for PTSD with a history of childhood abuse, while the major alleles, T allele of *rs3800373* and C allele of *rs1360780*, represent risk alleles for comorbid alcohol dependence and PTSD onset [42]. However, correlations between the risk allele of *FKBP5* and onset of PTSD have been inconsistent, and no direct research has investigated *FKBP5* and its role in alcohol consumption.

In the current study, we utilize the *Fkbp5* KO mouse model to address the functional relevance of *Fkbp5* in alcohol consumption and to map *Fkbp5* gene expression in brain. Furthermore, we extend the mouse findings by determining associations between *FKBP5* SNPs and alcohol drinking behaviors in humans, an effort critical for future translational research. Taken together, our findings are the first to demonstrate a role for *Fkbp5* in the regulation of alcohol drinking in both mice and humans.

## 2. Results

### 2.1. Alcohol Consumption Is Increased in Fkbp5^−/−^ Mice

**Figure 1:**
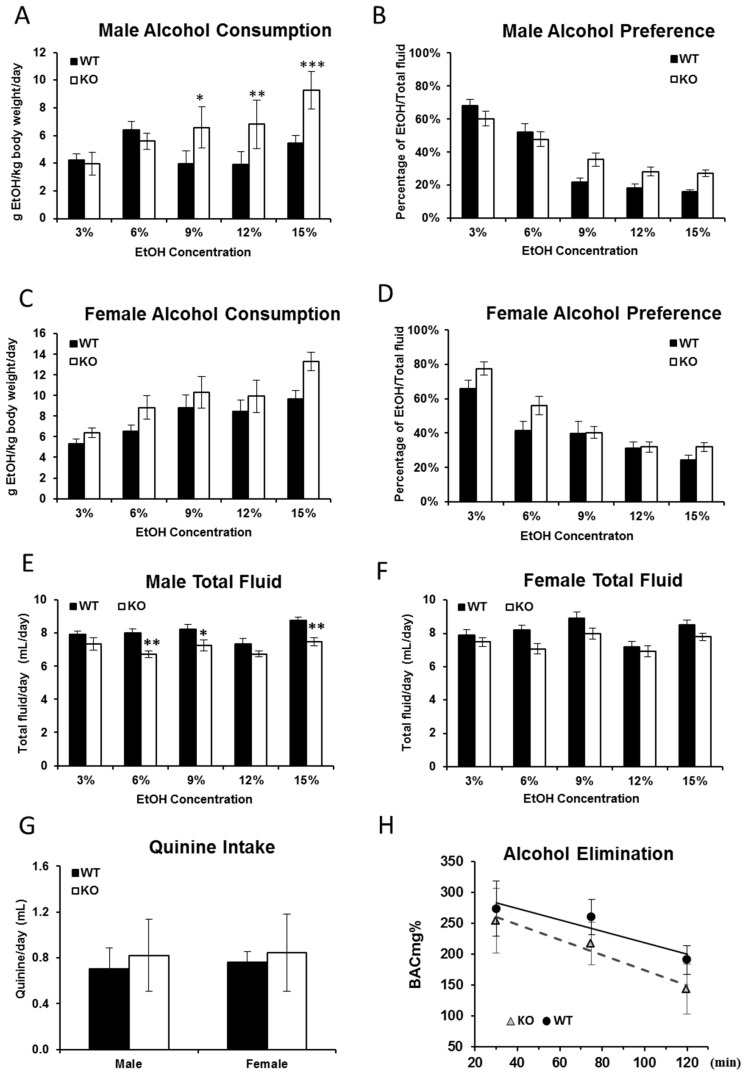
EtOH consumption and preference, quinine consumption and EtOH metabolism in *Fkbp5* KO and WT mice. A significant increase in alcohol consumption (**A**) in male mice was observed in the KO mice at 9%, 12% and 15% EtOH concentrations when compared to WT mice. No significant increases in alcohol consumption (**C**) in female mice or preference (**B**,**D**) in either sex were observed. Lower total fluid consumption was observed (**E**) in male mice, but not (**F**) in female mice during the EtOH consumption test. KO and WT mice did not differ in quinine consumption (**G**). No differences in alcohol metabolism were found between KO and WT mice (**H**). *, *p* < 0.05, **, *p* < 0.01, ***, *p* < 0.001.

Studies suggest that alcohol consumption is directly affected by circulating corticoid and GR availability [12] and that *FKBP5* plays a role in GR activation and *HPA* axis regulation [21]. In order to ascertain whether alcohol consumption was affected by *Fkbp5* gene knockout, a series of drinking tests were performed in both male and female adult mice. In the established alcohol drinking test protocol, two indices were calculated including alcohol consumption (g EtOH/kg body weight/day) and alcohol preference (percentage of EtOH/total fluid, *v*/*v*). A significant genotypic effect was observed on male alcohol consumption using repeated measures two-way ANOVA with *F* (1,47) = 9.27, *p* = 0.0038. Sidak’s multiple comparisons post hoc test revealed significant differences at 9% (*p* = 0.0226), 12% (*p* = 0.0081) and 15% (*p* = 0.0002) (Figure 1A). However, while repeated measures two-way ANOVA revealed significant differences due to ethanol concentration (*p* < 0.0001), no genotypic effects were observed in female alcohol consumption or in ethanol preference of either sex (Figure 1B–D). During the drinking tests, the body weight was measured twice per week. No significant changes in body weight were detected within genotype, regardless of sex. When different concentrations of alcohol were presented with water, the total fluid intake (volume of alcohol plus water) of KO mice was lower than WT in general, but only in males, this difference found to be significant via repeated measures two-way ANOVA with *F* (1,47) = 11.07, *p* = 0.0017. Sidak’s multiple comparisons post hoc test revealed significant differences at 6% (*p* = 0.0043), 9% (*p* = 0.0495) and 15% (*p* = 0.0032) (Figure 1E,F). Taken together, the current results suggest that deficiency of *Fkbp5* can enhance EtOH intake, at least in males.

To exclude the possibility that taste sensitivity may have been influenced by *Fkbp5* KO, animals were tested for quinine consumption (Figure 1G), but no significant difference was observed between KO and WT mice of either sex. Finally, we determined whether the observed differences in alcohol consumption might be due to differences in the rate of alcohol metabolism between genotypes. An ethanol dose of 2.0 g/kg body weight was injected intraperitoneally (IP), and the EtOH elimination rate was assessed. Regardless of sex, no significant difference in alcohol elimination rate was observed between *Fkbp5* KO (1.2 ± 0.13 mg% EtOH per min) and WT mice (0.9 ± 0.23 mg% EtOH per min) (Figure 1H).

### 2.2. Blood Alcohol Concentration Is Higher in KO than WT Mice

Blood alcohol concentration (BAC) was measured after 3 h of limited access to 15% EtOH. *Fkbp5* KO mice consumed more alcohol than WT mice (mean = 2.24 g EtOH/kg body weight for KO vs. 0. 94 g/kg for WT), resulting in higher BACs for KO mice compared to WT mice (mean = 53 ± 1.3 mg% EtOH in KO vs. 25.9 ± 1.92 mg% EtOH in WT).

### 2.3. The Fkbp5 Gene Is Highly Expressed in Brain Regions Important for the Stress Response

**Figure 2:**
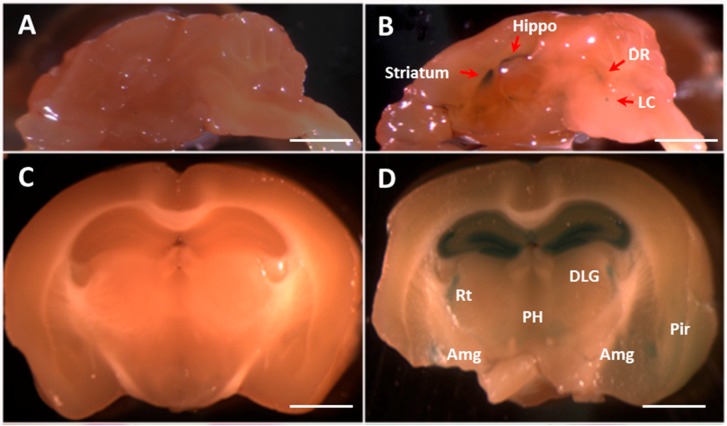

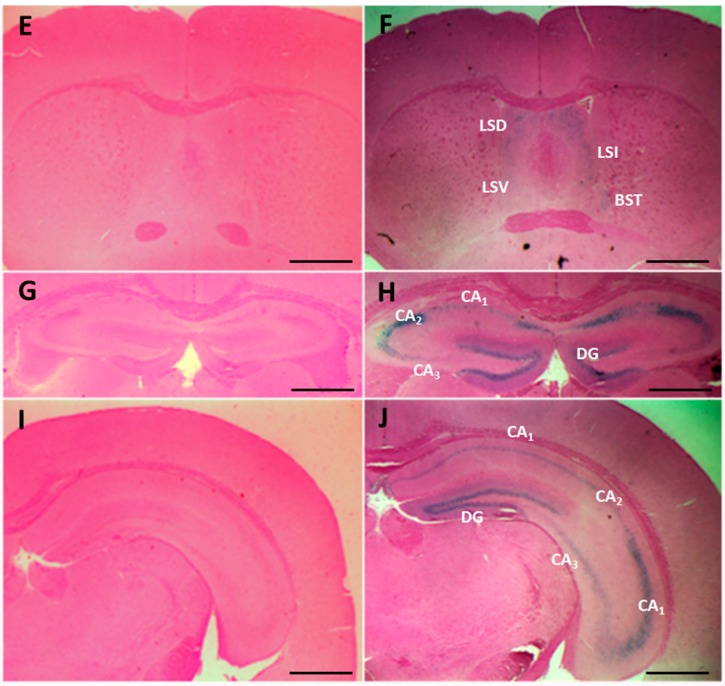
FKBP5 expression in the brain of WT and KO mice. Whole brain staining using the *LacZ* reporter gene. For each pairing of photomicrographs, the left panel is the WT control sample and the right panel is the KO sample. *Fkbp5* gene expression was observed in the brain regions of mice at one month (**A**,**B**) and four months (**C**–**J**) of age. Whole brain staining for *LacZ* in fresh tissue was performed (**A**–**D**) followed by hematoxylin and eosin staining in the four-month-old sample (**E**–**J**). Hippo (hippocampus), DR (dorsal raphe nucleus), LC (locus coeruleus), DLG (dorsal lateral geniculate nucleus (nu)), Rt (reticular thalamic (nu)), PH (posterior hypothalamic area), Amg (amygdala), Pir (piriform cortex), LSD (lateral septal nucleus, dorsal part), LSI (intermediate part), LSV (ventral part), BST (bed nucleus of the stria terminalis), CA (field CA of Ammon’s horn) and DG (dentate gyrus). Scale bars (**A**,**B**) = 2 mm, scale bars (**C**,**D**) = 1.5 mm, scale bars (**E**–**J**) = 1 mm.

*FKBP5* has been found to have reduced expression in individuals with PTSD [45]. *Fkbp5*-deficient mice were generated using a gene-trapping approach [46] in which the *LacZ* reporter gene was inserted. Previous studies have also shown that the murine *Fkbp5* gene is expressed in various tissues, including brain and peripheral tissue. We therefore charted the *Fkbp5* gene expression pattern in KO mice by staining for the *LacZ* gene product. In the brains of one-month-old *Fkbp5* KO mice, *LacZ* staining revealed *Fkbp5* expression in the hippocampus, striatum, dorsal raphe (DR) and locus coeruleus (LC) (Figure 2B). In four-month-old *Fkbp5* KO mice, *LacZ* staining was observed in the hippocampus and additional brain regions, such as amygdala (Figure 2D). Whole brain *LacZ* staining followed by sectioning and eosin red staining in four-month-old KO mice revealed that *Fkpb5* was highly expressed in the lateral septum, which includes lateral septal nuclei dorsal (LSD), ventral (LSV) and intermediate (LSI) (Figure 2F); bed nucleus of the striatum terminalis (BST) (Figure 2F); and hippocampus (Figure 2H,J). In the four-month-old hippocampus, *LacZ* staining suggested that *Fkbp5* is highly expressed in CA1, CA2, CA3 and the dentate gyrus (DG) (Figure 2D,H,J). Consistent with the absence of the *LacZ* reporter gene, WT control mice did not exhibit any *LacZ* staining (Figure 2A,C,E,G,I).

### 2.4. Basal Corticosterone Is Not Different Between Fkbp5^−/−^ and WT Mice

**Figure 3:**
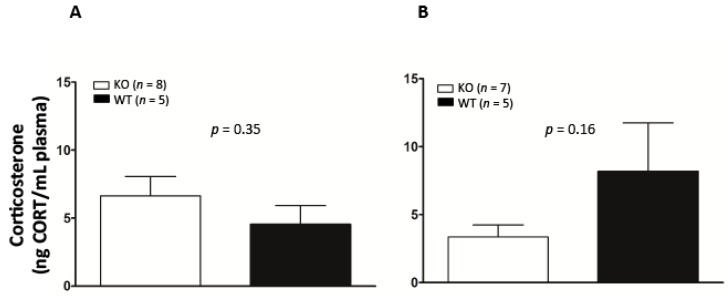
Basal serum corticosterone (CORT) levels in male (**A**) and female (**B**) WT and KO mice. No significant difference was found between genotypes.

Multiple lines of evidence demonstrate that Fkbp5 expression responds to CORT treatment [32,47]. Basal CORT was measured in blood samples of KO and WT mice to determine if there was a genotype and sex difference. No significant differences between KO and WT were detected by ANOVA (genotype × sex) (interaction term: *F* (1,21) = 3.4, *p* = 0.08) (Figure 3). However, there is trend of lower baseline CORT in female KO compared to WT.

### 2.5. SNPs in FKBP5 Are Associated with Alcohol Drinking Behavior in Humans

Genotype frequency and allele frequency are shown in Table 1 split by ethnic group (including all 1162 participants regardless of drinking status). Genotype distributions were in Hardy-Weinberg equilibrium (a principle that the genetic variation in a population will remain constant from one generation to the next without disturbing factors) for all three ethnic groups (*p* > 0.40). Our results were consistent with previous findings that have found the two SNPs in the *FKBP5* gene (*rs3800373* in the 3’UTR and *rs1360780* in intron 2) to be highly linked [38]. The two SNPs were linked among 92%–97% of all subjects studied (Table 1).

Significant associations of both SNPs with alcohol measures were found in Chinese and Koreans, but not in Caucasians (Table 2) for raw means and standard deviations and regression statistics using transformed variables. For the *rs1360780* SNP, Chinese with the *CC* genotype had significantly higher scores than those with *CT*/*TT* genotypes on average quantity, binge drinking episodes, lifetime maximum drinks and lifetime alcohol use disorder (AUD) symptoms, although lifetime maximum drinks was only significant in Chinese men (12.0 vs. 7.2, *F* = 9.32, *p* = 0.003, *R*^2^ change = 0.049). Koreans with the *CC* genotype also had significantly higher scores on AUD symptoms than Koreans with *CT*/*TT* genotypes. For the *FKBP5* 3’-UTR *rs3800373* SNP, Chinese with the *TT* genotype had higher scores on lifetime maximum drinks and AUD symptoms than the *TG*/*GG* genotypes; again, lifetime maximum drinks was only significant in Chinese men (11.8 vs. 7.4, *F* = 13.37, *p* < 0.001, *R*^2^ change = 0.069). Koreans with the *TT* genotype also had a greater number of lifetime AUD symptoms than Koreans with *TG*/*GG* genotypes. No gene-binge drinking interaction terms were significant.

## 3. Discussion

The current studies investigated the role that *Fkbp5* plays in alcohol drinking in both mice and humans. Compared to WT mice, *Fkbp5* KO mice exhibited increases in alcohol. These increases were not attributed to sensitivity to a bitter-tasting solution or to differences in the rate of alcohol metabolism. Relevant to its function in the stress response, *Fkbp5* was found to be expressed in brain regions that are important in the stress response. Consistent with a previous study [26], basal differences in CORT levels were not observed between genotypes in male mice; although a trend toward lower basal CORT in female *Fkbp5*-deficient mice can be seen in Figure 3, consistent with those findings [26]. Finally, *FKBP5* SNPs were associated with alcohol consumption in Asians, but not Caucasians. This is the first study to demonstrate a role for the *Fkbp5* gene in regulating alcohol consumption in both mouse and human samples.

### 3.1. FKBP5 Is Associated with Psychiatric Disease Including Alcohol Use Disorder

Previous research has focused on *FKBP5* and its association with various psychiatric disorders [21,41,48,49]. This study demonstrated that the gene expression of *Fkbp5* in the mouse plays at least a partial role in the regulation of alcohol drinking; KO mice with no expression of *Fkbp5* consumed more alcohol. Even though we do not have RNA available for *FKBP5* gene expression measurement in humans, the results from previous research also support the hypothesis that low *FKBP5* expression is associated with greater alcohol consumption. Previous research has shown that *rs1360780* (*CC*) homozygous subjects exhibit decreased *FKBP5* mRNA expression compared to (*TT*) homozygous subjects [38]. Consistent with the assumption that subjects with *CC* genotypes also display lower *FKBP5* mRNA expression than subjects with *CT* or *TT* genotypes, we found that Chinese and Koreans with *CC* had higher scores on alcohol-related variables than *CT*/*TT* carriers.

*FKBP5* SNPs and gene expression levels are associated with the onset of PTSD and anxiety disorder in humans [20,34]. These SNPs are also associated with altered response to antidepressants [39,40,41]. Particularly, heterozygous and homozygous carriers of the *rs3800373* (*TG* and *GG*) or *rs1360780* (*CT* and *TT*) variants are more likely to respond to antidepressant drugs [44]. A recent study also indicated that aged (>50 years) *T* allele carriers of *rs1360780* showed significantly higher induction of *FKBP5* mRNA expression by glucocorticoids in peripheral blood mononuclear cells [50]. Additionally, the effect of increasing severity of childhood abuse on the resultant level of adult PTSD appears to be carried by a subset of subjects with more common alleles of *FKBP5* [34]. These findings suggest that the *G* allele of *rs3800373* and the T allele of *rs1360780* (minor alleles) may represent protective alleles for PTSD with a history of childhood abuse, while the major alleles, the T allele of *rs3800373* and the *C* allele of *rs1360780*, represent risk alleles. In agreement with previous studies, these major alleles are associated with a higher risk for comorbid alcohol dependence and PTSD onset [42].

In our prior study, alcohol-dependent inpatients with the T allele of *rs3800373* had more severe withdrawal symptoms [5]. In this study, this major allele is associated with higher scores on alcohol-related variables. Interestingly, the *rs1360780* SNP is within an intronic region; a homologous region in rodent *Fkbp5* is highly conserved and has a functional hormone response element [51]. We speculate that *rs1360780* may be a functional SNP that regulates gene expression, which in turn alters alcohol drinking. In summary, the major alleles of *FKBP5* SNPs are associated with higher levels of alcohol consumption, more frequent consumption and a greater likelihood for alcohol-related problems in this study, as well as with withdrawal symptoms in prior studies [5]. In the future, genotyping *FKBP5* SNPs in heavy drinkers and the general population may explain the previous finding, which demonstrated that alcohol-dependent subjects had more withdrawal symptoms, higher alcohol intake and a higher maximum number of drinks compared to the general population [52]. *FKBP5* can be used as a biomarker for the diagnosis of alcohol use disorder.

This study demonstrates ethnic differences in the association of *FKBP5* SNPs with alcohol-related phenotypes. In Korean and Chinese individuals, significant associations were found between *FKBP5* SNPs and alcohol drinking-related variables, but no *FKBP5* gene effects were detected in Caucasian participants. Previous research has found a combinatorial effect of the *FKBP5* gene and childhood abuse on the risk for developing PTSD in African Americans, but not in European Americans [42]. Results of this study also indicated consistent findings across gender within each ethnicity with the exception of the association of *FKBP5* SNPs with lifetime maximum number of drinks in Chinese, which only reached significance in the men. This lack of significance in the Chinese female sample may be due to statistical power given the lower levels of maximum drinks in the women overall, with the trends being similar in both genders. In addition, our analyses did not indicate that recent heavier drinking was associated with a differential effect of the genes on lifetime heavy drinking or problems, further indicating the consistency of the findings within each ethnic group despite differences between the ethnic groups. Future research should continue to investigate gender and ethnic group differences in the associations of these SNPs with alcohol-related behaviors to better understand their relationships in those with different allele prevalence and consumption patterns, including those with more severe alcohol-related problems.

### 3.2. No Basal Difference in CORT Level, but Fkbp5 Expressed in the Brain Regions Involving Stress Response

Previous studies have indicated a correlation between alcohol consumption, glucocorticoid levels and GR activity [4,14,53]. Animal studies show that circulating levels of CORT rise after stress in *Fkbp5* KO mice [54] and prenatally stressed rats [27]. Consistent with a previous study [26], we did not find basal differences in CORT levels between genotypes in male mice; although, a trend toward lower basal CORT in female *Fkbp5*-deficient mice can be seen in Figure 3. Based on our research and that of others, it is clear that eliminating *Fkbp5* does not affect basal CORT. Given the tantalizing result that *Fkbp5* is expressed in the brain regions that are relevant to stress response, it will be very interesting to study how stress affects CORT levels in *Fkbp5* KO mice.

In the periphery, as well as the CNS, *Fkbp5* plays an important role in the stress response. Research has shown *Fkbp5* gene expression in the adult brain [24,55] and in mice as young as three months [56]. The current study revealed *Fkbp5* expression in the brain of one-month-old mice. Brain regions with high expression of *Fkbp5* have been implicated in both the stress response and the development of alcohol dependence [57,58]. *Fkbp5* expression is induced in the brain by stress [24,27], alcohol [28,29] and other drugs [30]. Our ongoing studies aim to understand how stress and alcohol affect *Fkbp5* expression in the brain and circulating CORT levels.

The limitation of this research is that because *Fkbp5* is a stress response gene, other stressors could be confounders; for example, we did not include smoking as a covariate in our analyses and only assessed current consumption patterns and lifetime alcohol problems in these analyses.

## 4. Materials and Methods

### 4.1. Animal and Human Subjects

All experimental protocols were reviewed and approved by the Animal Care and Use Committees at the Indiana University School of Medicine (protocol #DS0000871R, date of approval 4/24/2013), Purdue University (#1112000327, 10/11/2013), and the Institute of Laboratory Animal Science of Peking Union Medical College (#ILAS-PG-2014-013). These protocols were carried out in accordance with the NIH Guide for the Care and Use of Laboratory Animals. As described in a previous paper, *Fkbp5* knockout (*Fkbp5*^−/−^) mice were generated using the gene trapping method [46]. *Fkbp5*^−/−^ and WT littermates were bred through heterozygous mating and were backcrossed with C57BL/6J inbred mice for at least 5 generations.

The human study (#041122, 9/2/2004; #080978, 5/22/2008; #100399, 2/25/2010) was approved by the University of California, San Diego (UCSD) Human Research Protections Program and had a Certificate of Confidentiality from the USA Department of Health and Human Services. UCSD college students of legal drinking age were recruited to participate through advertisements on campus. All participants provided written informed consent.

### 4.2. Alcohol Drinking Tests

Drinking experiments were repeated three times using different cohorts of animals, in all a total of 82 *Fkbp5^−/−^* (male, *n* = 46; female, *n* = 36) and 92 WT (male, *n* = 52; female, *n* = 40) mice. Fluid intake and body weight were measured every other day throughout each experiment. The average body weights of the mice were WT = 30 g and KO = 27 g for males and WT = 24 g and KO = 22 g for females. At 13 weeks of age, mice were habituated to drinking in their home cages for 6 days with two 25-mL graduated cylinders containing water. Following the habituation phase, mice were given 24-h access to water and alcohol (EtOH). The concentration of EtOH (*v*/*v*) was increased every four days as follows: 3%, 6%, 9%, 12% and 15%. Average alcohol consumption per day was calculated and corrected for individual differences in body weight (BW) (g EtOH/kg BW/day). Alcohol preference ratios were then calculated (EtOH/total fluid). To exclude the possibility that KO and WT mice differed in taste reactivity, animals were tested for quinine (0.5 µM) intake as published [59].

### 4.3. Alcohol Elimination Rate

At 12 weeks of age, the EtOH elimination rate was determined using a separate cohort of *Fkbp5^−/−^* and WT mice (4 male and 4 female mice of each genotype). An EtOH dose of 2.0 g/kg was injected intraperitoneally (IP). Tail blood was collected at 30, 75 and 120 min after IP injection, and plasma samples were spun down and stored at −80 °C until further analysis [60]. Plasma alcohol concentrations were measured using gas chromatography according to the instructions of the manufacturer (HP Agilent, Santa Clara, CA, USA).

### 4.4. Blood Alcohol Concentration after 3-h Limited Access

Mice (10 *Fkbp5^−/−^* and 10 WT mice of both sexes at 6 months of age) were maintained at reverse light cycle for 3 weeks before the experiment. The subjective dark period is the active time for rodents. In order to assess the blood EtOH levels achieved during the active period, intake volume of a 15% EtOH solution and subsequent blood EtOH concentrations were determined after a 3-h access period, occurring 3 h into the dark cycle. On the test day, a stacking design was used with 10-min intervals separating the presentation of EtOH to each mouse. Beginning at 7:00 a.m., the first animal received 15% EtOH, and tail blood was taken at 10:00 a.m., with the rest of the mice following in sequence at 10-min intervals. Blood was collected in an EDTA-coated tube and stored on ice until all samples were collected. After collection, blood samples were centrifuged at 1500× *g* for 10 min, and plasma was isolated for blood alcohol concentration measurement. The same GC platform was used as described above.

### 4.5. LacZ Staining

Age-matched *Fkbp5*^−/−^ (*n* = 6) and littermate control (*n* = 6) mice of both sexes were used for morphological and histological studies. *Fkbp5* gene expression in the whole brain was determined at 1 and 4 months of age. Whole brains were collected, and the *LacZ* gene product, β-galactosidase activity, was detected using X-gal [48,61]. After images were collected, the whole brain or brain slices were fixed in 10% neutral-buffered formalin. Brains were paraffin embedded, sectioned (5 µm) and stained with hematoxylin and eosin following standard protocols [62,63].

### 4.6. CORT Measurement

Basal corticosterone (CORT) levels were measured in mice. Blood samples were collected from the submandibular vein (male: KO = 8, WT = 5; female: KO = 7, WT = 5) between 1200 and 1300 (light cycle on at 700–1900). The concentration of CORT in the blood was determined using a competitive enzyme immunoassay kit from Assay Designs (Ann Arbor, MI, USA), as previously published [64]. Optical densities were read on a Multiskan^TM^ FC microplate reader (ThermoFisher Scientific Inc., Waltham, MA, USA). Standards and plasma samples were analyzed in duplicate. CORT concentrations were interpolated from standard curves generated using a four-parameter logistic curve fitting program (GraphPad Inc., San Diego, CA, USA).

### 4.7. Human Sample Phenotypes

Participants were students at UCSD (*n* = 1162) 21–26 years of age (*M* = 22.0, *SD* = 1.34), with an average of 15.1 years of education (*SD* = 0.89), who reported that all 4 of their grandparents were entirely of Chinese (*n* = 360, 48% female), Korean (*n* = 343, 50% female) or Caucasian (*n* = 449, 48% female) ethnicity. Participants completed the time-line follow-back measure to evaluate alcohol consumption for the preceding 90 days [65,66]. A standard drink was defined as 12 oz (355 mL) of beer, 5 oz (150 mL) of wine or 1.5 oz (45 mL) of hard liquor. These amounts are the equivalent of approximately 14 g of pure ethanol. Participants also completed the Semi-Structured Assessment for the Genetics of Alcohol use disorder [67,68] with a trained research interviewer. During a portion of this interview, participants recounted the maximum number of drinks ever consumed in a 24-h period and were assessed for the 11 DSM-IV alcohol abuse and dependence symptoms (range 0–11; American Psychological Association, 1994) [69]. Five alcohol-related variables were calculated from these assessments: (1) average quantity of drinking during the previous three months (standard drinks/occasion); (2) average frequency of drinking during the previous three months (days/month); (3) number of binge drinking episodes (four or more drinks on an occasion for women and five or more on an occasion for men) during the previous three months; (4) lifetime maximum number of alcohol drinks ever consumed in a 24-h period; and (5) number of lifetime alcohol use disorder (AUD) symptoms (alcohol abuse and dependence). Lifetime non-drinkers (defined as never having had a standard drink of alcohol) were excluded (*n* = 30).

### 4.8. Human Sample Genotyping

Blood samples were collected by fingertip puncture and delivered to the Genomics and Bioinformatics Core of Indiana Alcohol Research Center for genotyping. Genomic DNA was isolated by the “HotSHOT” method [70], and TaqMan probes were used for allelic discrimination (Life Technologies, Foster City, CA, USA). Genotyping procedures were reported previously [71]. Two SNPs, *rs1360780* (major/minor allele, *C*/*T*) within intron 2 and *rs3800373* (*T*/*G*) in the 3’UTR, were selected for genotyping. The two SNPs selected comprise one single large linkage disequilibrium (LD) block based on previous LD structure analysis [38].

### 4.9. Statistical Analysis

Total fluid intake and alcohol consumption and preference differences at tested EtOH concentrations between genotypes were assessed via 2-way repeated measures analysis of variance (ANOVA) with Sidak’s test for multiple comparisons. Differences in quinine intake and CORT data were analyzed using Student’s *t*-test.

In humans, data were analyzed using linear regressions. All alcohol variables were log transformed to account for the non-normality of the distribution of raw scores. Based on the genotypic distributions (Table 1) and consistent with prior studies [44], we dichotomized the genotype groups, such that *CT* and *TT* were combined for the *FKBP5* intron SNP (*rs1360780*) and *TG* and *GG* were combined for the *FKBP5* 3’-UTR SNP (*rs3800373*). Because of the strong association of alcohol metabolizing genes (e.g., *ALDH2, ADH1B*) with alcohol-related behaviors [72], we co-varied for these two genotypes (dichotomized variables for those with and without the variant ALDH2*2 and ADH1B*2 alleles) by entering them in linear regressions as the first step prior to entering the *FKBP5* gene in the second step. Note that the ALDH2*2 allele was present only in the Chinese and Korean groups, whereas the ADH1B*2 was present in all three ethnic groups.

We tested for gender differences in the relationship of the *FKBP5* SNPs with alcohol-related behaviors by including gene-gender interaction terms in the regression models. For any interaction term with a *p* < 0.10, we then examined the relationship of the gene with the alcohol-related variable in each gender separately. We used a similar approach to examine the possibility that current heavy alcohol consumption might differentially affect the association of the genes with lifetime heavy drinking and AUD symptoms; in these analyses, we included interaction terms of the genes with having binged in the past 3 months.

## 5. Conclusions

Despite these limitations, our study demonstrates that *FKBP5* is associated with alcohol consumption phenotypes in mice and in humans of Asian, but not Caucasian, descent. The current study has established the foundation for future studies investigating the potential role of *FKBP5* in responses to acute and chronic drinking, alcohol withdrawal symptoms and the interplay of stress and *Fkbp5* expression on the development of alcohol dependence. *FKBP5* may well be an interesting therapeutic target for the prevention and treatment of stress-related alcohol drinking behavior.

## Figures and Tables

**Table 1:** *FKBP5* genotype distributions and Hardy–Weinberg equilibrium (HWE) values for each ethnic group. The chi-square statistic (X^2^) is used to test if the allele frequencies are in HWE for each ethnic group, that is they are consistent with the expected distribution for the general population. The significance tests (*p*-values), all being >0.05, show that the alleles are in HWE, indicating no deviation from the expected distribution of alleles in the population and no bias in our samples.

SNP	*FKBP5* Intron (*rs1360780*)					*FKBP5* 3’-UTR (*rs3800373*)				
	Genotype Distribution			HWE		Genotype Distribution			HWE	
Ethnicity	*CC*	*CT*	*TT*	X^2^	*p*	*TT*	*TG*	*GG*	X^2^	*p*
Chinese	190 (53%)	141 (39%)	29 (8%)	0.2	0.69	192 (53%)	138 (38%)	29 (8%)	0.4	0.55
Korean	195 (57%)	131 (38%)	17 (5%)	0.7	0.40	205 (60%)	123 (36%)	15 (4%)	0.4	0.52
Caucasian	226 (50%)	189 (42%)	34 (8%)	0.4	0.52	240 (46%)	175 (33%)	33 (6%)	0.0	0.89

**Table 2:** Alcohol-related variables in Chinese, Korean and Caucasian college students for two *FKBP5* genotypes after co-varying for ALDH2*2 and ADH1B*2.

SNP	*FKBP5* Intron (*rs1360780*)					*FKBP51* UTR (*rs3800373*)				
Genotype	*CC*	*CT*/*TT*	–	–	–	*TT*	*TG*/*GG*	–	–	–
Statistic	*M* (*SD*)	*M* (*SD*)	*F*	*p*	Change in *R*^2^	*M* (*SD*)	*M* (*SD*)	*F*	*p*	Change in *R*^2^
Chinese	*n* = 180	*n* = 160	–	–	–	*n* = 183	*n* = 157	–	–	–
Quantity	3.8 (6.02)	2.9 (1.52)	2.13	**0.034**	0.013	3.3 (2.11)	2.9 (1.53)	1.39	0.164	0.005
Frequency	10.9 (11.31)	9.7 (9.61)	0.69	0.491	0.001	10.7 (11.30)	9.8 (9.64)	0.34	0.731	0.001
Binges	3.3 (5.20)	2.8 (4.70)	1.96	0.051	0.010	3.2 (5.17)	2.8 (4.74)	1.60	0.110	0.007
Max drinks	9.1 (8.54)	6.3 (5.21)	3.69	**<0.001**	0.037	8.7 (8.46)	6.4 (5.21)	2.84	**0.005**	0.022
AUD symptoms	1.4 (1.00)	1.2 (0.65)	2.33	**0.021**	0.015	1.4 (0.97)	1.2 (0.65)	1.99	**0.048**	0.012
Korean	*n* = 188	*n* = 146	–	–	–	*n* = 198	*n* = 136	–	–	–
Quantity	4.2 (2.48)	3.8 (2.21)	1.18	0.238	0.004	4.1 (2.44)	3.9 (2.20)	0.33	0.741	0.001
Frequency	15.1 (14.89)	12.2 (11.99)	1.63	0.105	0.008	14.8 (15.01)	12.5 (11.97)	0.89	0.374	0.002
Binges	6.1 (8.17)	5.2 (7.88)	1.37	0.172	0.006	5.8 (7.99)	5.4 (8.03)	0.58	0.560	0.001
Max drinks	12.1 (9.89)	10.4 (7.32)	1.57	0.118	0.007	11.7 (9.73)	10.7 (7.20)	0.52	0.603	0.000
AUD symptoms	2.1 (1.77)	1.7 (1.29)	2.40	**0.017**	0.017	2.0 (1.72)	1.7 (1.31)	1.95	0.052	0.011
Caucasian	*n* = 217	*n* = 220	–	–	–	*n* = 203	*n* = 235	–	–	–
Quantity	3.1 (1.86)	3.3 (2.24)	0.21	0.65	0.000	3.2 (1.98)	3.2 (2.20)	0.00	0.98	0.000
Frequency	24.5 (18.80)	24.2 (17.19)	0.04	0.84	0.000	24.2 (18.40)	24.1 (17.42)	0.08	0.77	0.000
Binges	8.2 (12.16)	8.0 (10.51)	0.20	0.65	0.000	8.2 (12.28)	8.0 (10.63)	0.05	0.82	0.000
Max drinks	13.9 (10.13)	14.2 (11.83)	0.23	0.63	0.001	14.0 (10.35)	13.9 (11.42)	0.06	0.81	0.000
AUD symptoms	1.0 (1.71)	1.0 (1.61)	0.10	0.76	0.000	1.0 (1.75)	1.0 (1.58)	0.63	0.43	0.001

Raw scores reported for means and standard deviations. In the analyses, alcohol variables were log transformed to adjust for right-tailed distributions. ALDH2*2 and ADH1B*2 were covaried in all analyses, but no Caucasian possessed an ALDH2*2 allele. AUD, alcohol use disorder. Bolded *p*-value indicated statistically significant at *p* < 0.05.
